# Reducing seed waste and increasing value of dynamic intraoperative implantation of Pd‐103 seeds in prostate brachytherapy

**DOI:** 10.1002/acm2.12404

**Published:** 2018-07-13

**Authors:** Peter K. Taylor, Adam C. Riegel

**Affiliations:** ^1^ Northwell Health System Department of Radiation Medicine Center for Advanced Medicine Lake Success NY USA; ^2^ Hofstra Northwell School of Medicine Hempstead NY USA

**Keywords:** brachytherapy, nomogram, palladium‐103, prostate seed implant

## Abstract

Several nomograms exist for ordering palladium‐103 seeds for permanent prostate seed implants (PSI). Excess seeds from PSIs pose additional radiation safety risks and increase the cost of care. This study compared five nomograms to clinical data from dynamic modified‐peripheral intraoperative PSI to determine (a) the cause of excess seeds and (b) the optimal nomogram for our institution. Pre‐ and intraoperative patient data were collected for monotherapy PSIs and compiled into a clinical database. All patients were prescribed 125 Gy with dose coverage of D90% = 100% to the planning target volume (PTV) using ^103^Pd seeds with mean air‐kerma strength ($\bar{S_{K}}$) of 2 U. Seeds were ordered based upon an in‐house nomogram as a function of preoperative prostate volume and prescription dose. Preoperative prostate volume was assessed with transrectal ultrasound. If any of the following four conditions were not met: (a) preoperative volume = intraoperative volume, (b) D90% = 100%, (c) $\bar{S_{K}}=2U$, and (d) seed ordering matched the in‐house nomogram, then a normalization factor was applied to the number of seeds used intraoperatively to meet all four conditions. Four published nomograms, an in‐house nomogram, and the normalized number of implanted seeds for each patient were plotted against intraoperative prostate volume. Of the 226 patients, 223 had excess seeds at the completion of their PSI. On average, 25.7 ± 9.9% of ordered seeds were not implanted. Excess seeds were separated into two categories, accounted‐for excess, determined by the four normalization factors, and residual excess, assumed to be due to overordering. The upper 99.9% CI linear fit of the normalized clinical data plus a 5% “cushion” may provide a more reasonable nomogram for ^103^Pd seed ordering for our institution. Nomograms customized for individual institutions may reduce seed waste, thereby reducing radiation safety risks and increasing the value of prostate brachytherapy.

## 1 INTRODUCTION

Many treatment options, such as radical prostatectomy, external beam radiation therapy (EBRT), brachytherapy, hormonal treatment, and active surveillance, exist for patients with prostate cancer.^1^ Transperineal interstitial permanent prostate brachytherapy (TIPPB) for early stage prostate cancer is an outpatient procedure involving insertion of radioactive seeds into the prostate under transrectal ultrasound guidance.^1^ Current radioisotopes used in TIPPB include ^125^I, ^103^Pd, and ^131^Cs.^1^, ^2^, ^3^, ^4^ At our institution, ^103^Pd seeds are implanted using a dynamic intraoperative technique^1^, ^2^, ^3^ and the workflow for seed ordering, implantation, and postimplant dosimetry is similar to that described in AAPM TG‐64.^2^ The workflow is as follows: (a) ^103^Pd seeds are ordered using a nomogram based on preoperative prostate volume, (b) the ^103^Pd seeds are independently assayed, (c) the ^103^Pd seeds are implanted into the prostate under transrectal ultrasound guidance, (d) postoperative excess ^103^Pd seeds (if applicable) are counted and disposed of by the medical physicist, and (e) postoperative dosimetry is performed approximately 4 weeks later.

Preoperative imaging of the patient's prostate is used to determine the number of ^103^Pd seeds to be ordered. At our institution, a nomogram comparing total recommended air‐kerma strength (U) and prostate volume (cm^3^) is used to determine the recommended total air‐kerma strength for the implant. The number of required seeds is found by dividing the total air‐kerma strength by the requested air‐kerma strength per seed. In our clinical experience, we have often found that there are a substantial number of excess ^103^Pd seeds after prostate seed implants. Reducing the number of unused seeds can reduce radiation safety risks, reduce cost, and increase the value of prostate brachytherapy in the context of value‐based medicine, defined as, “the practice of medicine incorporating the highest level of evidence‐based data with the patient‐perceived value conferred by healthcare interventions for the resources expended.”^5^ The purpose of this study was, (a) determine the cause of excess ^103^Pd seeds in dynamic intraoperative TIPPB and (b) determine the optimal nomogram for our institution.

## 2 MATERIALS AND METHODS

Prostate cancer patients who received monotherapy using ^103^Pd to a dose of 125 Gy were retrospectively included in the chart review. Relevant dosimetric and volumetric pre‐, intra‐, and postoperative measurements were compiled into a clinical database.

### 2.1 Excess seeds

The clinical database was used to determine the number of excess seeds for each patient. The number of excess seeds (N_XS_) is defined in eq. (1).

(1) $$N_{\mathrm{XS}}=\left(number\;of\;seeds\;ordered\right)-\left(number\;of\;seeds\;used\;intra-operatively\right)$$

The total number of excess seeds can be separated into two categories, accounted‐for excess (A_XS_) and residual excess (R_XS_). The quantity A_XS_ is equal to the sum of the number of excess seeds remaining after an implant (*n*) which can be attributed to a specific reason (*i*) [eq. (2)]. The value of $n_{i}$ can be positive or negative.

(2) $$A_{\mathrm{XS}}=\sum_{i}n_{i}$$

Excess seeds that could not be attributed to a specific cause were considered “residual.” The quantity R_XS_ is equal to the number of excess seeds less the number of accounted‐for excess seeds [eq. (3)].

(3) $$R_{\mathrm{XS}}=N_{\mathrm{XS}}-A_{\mathrm{XS}}$$

We identified four potential causes of excess seeds which are summarized in Table 1 and described in greater detail below. The value $n_{\mathrm{vol}}$ was calculated by taking the difference between the numbers of seeds which should have been ordered for each respective volume (preoperative and intraoperative) based upon the in‐house nomogram. If preoperative prostate volume was greater than intraoperative prostate volume, additional, unnecessary seeds were ordered, i.e., $n_{\mathrm{vol}}>$. If the intraoperative prostate volume was greater than the preoperative volume, more seeds were used than anticipated, which lowered the expected number of excess seeds at the end of the procedure, i.e., $n_{\mathrm{vol}}$.

**Table 1:** Variables which contribute to deviations between the expected number of seeds used intraoperatively and actual number of seeds used intraoperatively

Reasons for excess seeds	Description	Number of excess seeds due to reason (i)
Change in prostate volume	Preoperative prostate volume ≠ intraoperative prostate volume	$n_{\mathrm{vol}}$
Intraoperative D90%	Intraoperative D90% ≠ 100% (i.e., a “hot” or “cold” implant)	$n_{D90\%}$
Mean air‐kerma strength	Mean air‐kerma strength (*S*_*K*_) ≠ 2 U per seed	$n_{U}$
Ordering	Number of ordered seeds deviated from nomogram	$n_{\mathrm{order}}$

The value $n_{\mathrm{order}}$ was the difference between what was ordered and what should have been ordered based on the in‐house nomogram and the preoperative prostate volume. The output of the nomogram is total recommended air‐kerma strength. When this value is converted into number of seeds using vendor specified air‐kerma strength per seed, it is not necessarily an integer. For simplicity in ordering and to insure enough seeds were present for the procedure, nomogram results were frequently rounded up to the nearest multiple of five. If more seeds were ordered than indicated by the nomogram, the result is a known excess compared to the nomogram, i.e., $n_{\mathrm{order}}$. If fewer seeds were ordered than indicated by the nomogram, the result is a known seed deficit, i.e., $n_{\mathrm{order}}$.

All patients were prescribed 125 Gy with dose coverage of D90% = 100% to the planning target volume (PTV). If the intraoperative D90% was less than 100%, i.e., a “cold implant,” then not enough seeds were used during the implant resulting in a known seed excess at the end of the procedure, i.e., $n_{D90\%}$. If the intraoperative D90% was greater than 100%, more seeds were used than anticipated which lowered the number of excess seeds at the end of the procedure, i.e., $n_{D90\%}$. If the mean air‐kerma strength ($\bar{S_{K}}$) for a batch of seeds was greater than 2 U per seed, fewer seeds were required to meet the prescription dose of D90% = 100% to the PTV resulting in a known seed excess, i.e., $n_{U}$. If the mean air‐kerma strength ($\bar{S_{K}}$) for a batch of seeds was less than 2 U per seed, a greater number of seeds were required to meet the prescription dose of D90% = 100% to the PTV resulting in a known seed deficit, i.e., $n_{U}$.

### 2.2 Clinical data normalization parameters

Once the cause of excess seeds was determined, the number of seeds used intraoperatively per patient was linearly normalized such that $\bar{S_{K}}=2\;U$ and the intraoperative D90% was equal to 100%. The normalized number of ^103^Pd seeds vs intraoperative prostate volume for each patient was plotted against intraoperative prostate volume and the upper 99.9% confidence interval of the patient data was generated.

## 3 RESULTS

Between 2010 and 2015, our institution treated 251 patients with TIPPB using ^103^Pd to a dose of 125 Gy. Pre‐, intra‐, and postoperative assessments were measured and compiled into a clinical database. For this study, exclusion criteria were: (a) an absolute difference greater than 20% between the intra‐ and postoperative D90% to the PTV (*n* = 20), (b) postoperative data were not available (*n* = 2), and (c) the combined modality therapy (external beam radiation therapy and TIPPB) nomogram was used to order seeds (*n* = 3). This left *n* = 226 patients in the study.

### 3.1 Excess seeds

Table 2 lists patient‐specific assessments for three sample patients. Table 3 shows the calculation performed using the assessments in Table 2 to determine *n*_*i*_ and subsequently N_XS_, A_XS_, and R_XS_. A negative sign in Table 3 indicates that variable contributed positively toward R_XS_, that is, there should have been more seeds at the end of the procedure due to that specific variable.

**Table 2:** Patient‐specific assessments for three sample patients

Patient‐specific assessments								
Patient	I	II	III	IV	V	VI	VII	VIII
	Preoperative volume (cm^3^)	# of seeds to order (Vol _pre‐op_)	# of seeds ordered	Intraoperative volume (cm^3^)	# of seeds to order (Vol_i‐o_)	# of seeds used	Mean *S*_*K*_ (U)	D90%_i‐o_
A	28.0	98	95	22.7	85	56	2.02	94.2
B	23.5	86	90	26.6	94	72	2.00	107.5
C	51.0	153	145	47.8	146	103	1.98	95.5

**Table 3:** Determination of residual‐excess (R_XS_) seeds for patients A, B, and C from Table 2

Determination of R_XS_							
Derivation using columns from Table 2	*N*_*XS*_	Number of excess seeds due to *i*				*A*_*XS*_	*R*_*XS*_
		*n*_*vol*_	*n*_order_	*n*_D90%_	*n*_*U*_		
	(III*–*VI)	(II–V)	(III–II)	$\left(\text{VI}\right)\left(\frac{100\%}{\text{VIII}}-1\right)$	$\left(\text{VI}\right)\left(1-\frac{2U}{\text{VII}}\right)$	$\sum_{i}n_{i}$	$N_{\mathrm{XS}}-\sum_{i}n_{i}$
A	39	13	−3	3.5	0.55	14.05	24.9
B	18	−8	4	−5.0	0.00	−9	27.0
C	42	7	−8	4.9	−1.04	2.9	39.1

**Figure 1:**
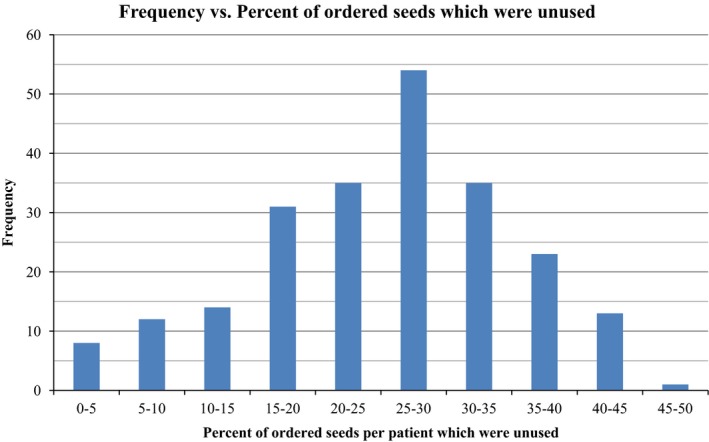
Histogram indicating the frequency of the percent of ordered seeds which were unused per prostate seed implant procedure.

Of the 226 prostate seed implants, 98.6% (*n* = 223) had excess seeds. On average (±1 standard deviation), there were 29.2 ± 13.2 excess seeds after the completion of a prostate seed implant. On average, 25.7 ± 9.9% of ordered seeds were not implanted. The percentage of ordered seeds which were wasted is the quotient of the number of excess seeds to the number of seeds ordered (eq. (4)). Figure 1 shows the distribution of the percentage of ordered seeds which were wasted.

(4) $$\text{Percentage of ordered seeds which were wasted =}\;\frac{\#\;\text{of excess seeds}}{\#\;\text{of seeds ordered}}\times 100\%$$

We found that none of the four parameters identified above contributed substantially to seed excess. In the cases where the intraoperative volume was greater than preoperative volume (53% of implants, *n* = 117), more seeds were used than anticipated to accommodate the larger prostate. In the cases where the implant had a D90% > 100%, i.e., a “hot implant” (72% of implants, *n* = 159), more seeds were used than anticipated which should have contributed towards the final number of excess seeds. In the cases where the mean air‐kerma strength was greater than 2 U (43% of implants, *n* = 98), more seeds were used than anticipated which should have contributed towards the number of excess seeds. The value of $n_{i}$ was on average negative for each “*i*.” The consequence of ${\overset{¯}{n}}_{i}<0$ means the number of residual‐excess seeds was on average greater than the physical number of excess seeds. On average, 30.7 ± 7.1% of ordered seeds were R_XS_. Table 4 shows the average N_XS_, R_XS_, and *n*_*i*_.

**Table 4:** Average number of excess seeds (N_XS_), number of excess seeds due to reason (*i*), and number of residual‐excess (R_XS_) seeds

	$N_{XS}$	$n_{vol}$	$n_{order}$	$n_{D90\%}$	$n_{U}$	$R_{\mathrm{XS}}$
${\overset{¯}{n}}_{i}\pm 1\sigma_{i}$	29.2 ± 13.2	−0.6 ± 13.9	−1.1 ± 3.5	−3.4 ± 6.9	−0.1 ± 1.7	34.4 ± 9.3

### 3.2 Nomogram

**Figure 2:**
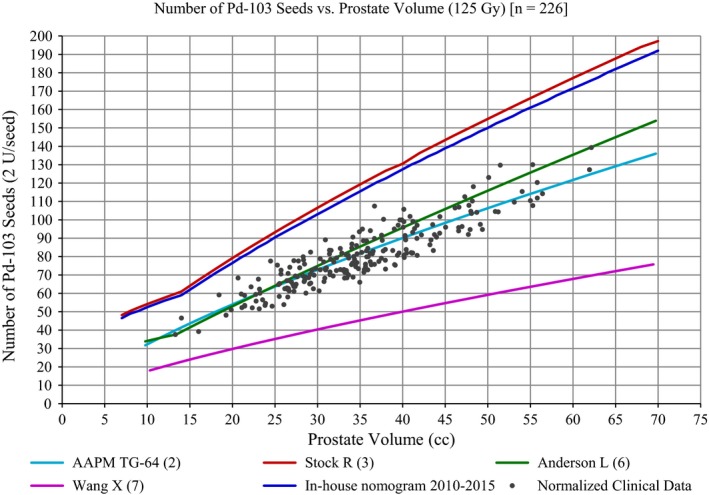
A comparison between four published nomograms, an in‐house nomogram, and the normalized clinical data (*n* = 226) for a prescription dose of 125 Gy.

The clinical data, after being normalized such that $\bar{S_{K}}=2U$ and D90% = 100%, was compared to five nomograms: AAPM TG‐64,^2^ Anderson^6^ (Memorial‐Sloan Kettering Cancer Center, 1993), Stock^3^ (Mt. Sinai, 1995), Wang^7^, and our in‐house nomogram currently in use since 2010 (Fig. 2). There were differences between prescription doses which required linear scaling, for example, Anderson uses a prescription dose of 115 Gy for ^103^Pd. If the AAPM TG‐64 or Anderson nomogram had been used at our institution, approximately 50% of normalized clinical cases would not have had sufficient seeds.

**Figure 3:**
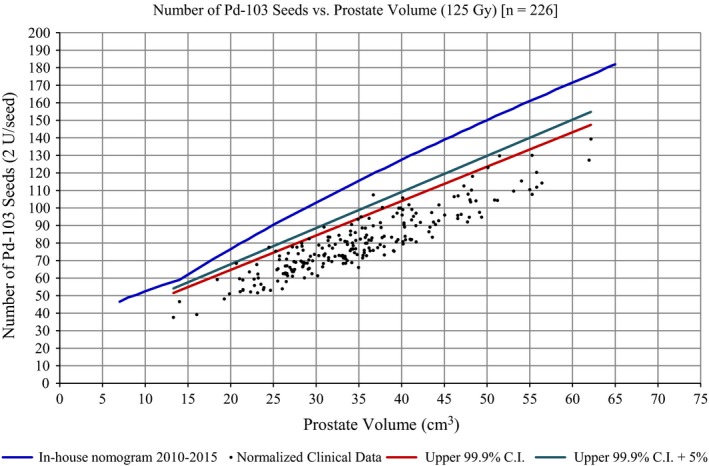
A comparison of the upper 99.9% confidence interval of the linear regression of the normalized clinical data compared to the in‐house nomogram.

Figure 3 is a plot of the upper 99.9% confidence interval (CI) of the normalized clinical data, the upper 99.9% CI plus a 5% “cushion,” the normalized clinical data, and the in‐house nomogram. If the upper 99.9% CI of the normalized clinical was used for ^103^Pd seed ordering, 12 out of 226 (5.3%) of normalized clinical cases would not have had sufficient seeds for the implant. The upper 99.9% CI plus a 5% overordering “cushion” would provide enough seeds for all but one patient.

## 4 DISCUSSION

Based upon the analysis of 226 patients spanning 6 yr, 25.7 ± 9.9% of ordered seeds were not implanted. As with most clinical procedures, there will be some discrepancy between what is planned and what occurs at the time of the procedure. We identified four variables in the prostate seed implant flow process which could be used to account for excess seeds, (a) variances between pre‐ and intraoperative prostate volume, (b) differences between what was ordered and what should have been ordered based upon the nomogram, (c) D90% not being equal to 100%, and (d) mean air‐kerma strength not being equal to 2 U. We found that none of these variables contributed substantially to the seed excess for our patient sample. The average number of excess seeds was 29.2 ± 13.2 and the average number of residual‐excess seeds was 34.4 ± 9.3. Overordering to assure a sufficient quantity of seeds during the prostate seed implant procedure may be contributing to R_XS_. If the upper 99.9% CI nomogram was used, 12 patients would not have had enough seeds for their procedure. If the upper 99.9% CI + 5% was used, one patient would not have had enough seeds for their procedure and we could potentially reduce the number of ordered seeds by 13%.

When the clinical data is compared to the other nomograms, two nomograms overestimate the number of required seeds (in‐house nomogram and Stock^3^), two nomograms lay within the range of the clinical data (AAPM TG‐64^2^ and Anderson^6^), and one underestimates the number of seeds required (Wang^7^). The disparity between nomograms could be attributed to their variety in formulation. The nomogram by Wang^7^ is a theoretical derivation where the prostate is modeled as a sphere and radioactivity is considered to be in continuous form. The Anderson nomogram was generated from matched peripheral dose of 13 ^103^Pd implants and 64 ^125^I implants evaluated as if ^103^Pd had been used.^6^

Our institution uses a modified peripheral loading technique, 3D transrectal ultrasound, and dynamic intraoperative delivery.^2^ This 3D method allows the prostate, PTV, organs at risk, needles, seeds, and dose distribution to be visualized in real time. It is possible the in‐house nomogram, which has been in use for nearly a decade, is not optimized for the current method of delivery. In the current work, we collected data spanning 6 yr from over 200 ^103^Pd patients to refine our seed ordering nomogram as per the American Brachytherapy Society^1^ and AAPM TG‐137^6^ recommendations for plan evaluation using D90% as a volumetric dose metric. Given the disparity between published nomograms, institution‐specific nomograms may provide a more efficient means to order and deliver TIPPB.

**Figure 4:**
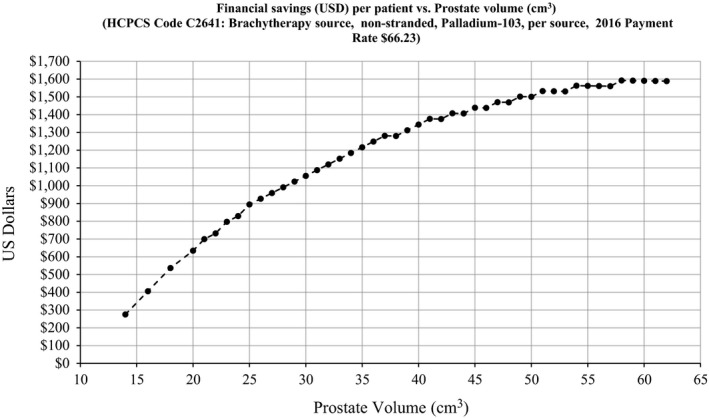
Financial savings per patient introduced by using a clinically derived nomogram for ^103^Pd brachytherapy prostate seed implant.

Reducing the number of excess seeds after a prostate seed implant is beneficial in improving safety but it also yields financial benefits. The savings in US Dollars vs prostate volume is demonstrated in Fig. 4. The savings was calculated by determining the difference in the number of seeds between the in‐house nomogram and the upper 99.9% CI + 5% “cushion.” This difference was multiplied by the 2016 Medicare payment rate of $66.23 per source (HCPCS Code C2641: Brachytherapy source, non‐stranded, Palladium‐103, per source). By applying the customized nomogram to our sample, we could potentially reduce costs by approximately $43,000 per year.

## 5 CONCLUSION

By collecting clinical data spanning 6 yr (2010–2015) and 226 patients, we found that over 85% of clinical cases wasted over 15% of ordered seeds. Substantial disparity between published nomograms was observed. The upper 99.9% CI of our clinical data plus a 5% “cushion” may provide a more reasonable nomogram for ^103^Pd seed ordering for our institution. Other institutions may benefit from a site‐specific nomogram. Reducing excess seeds will reduce exposure to staff, reduce the risk of accidental contamination, and reduce cost which may increase the value of prostate brachytherapy compared with other treatment modalities.

## AUTHORSHIP

All persons who meet authorship criteria are listed as authors, and all authors certify that they have participated sufficiently in the work to take public responsibility for the content, including participation in the concept, design, analysis, writing, or revision of the manuscript. Furthermore, each author certifies that this material or similar material has not been and will not be submitted to or published in any other publication.

## CONFLICTS OF INTEREST

The authors whose names are listed immediately below certify that they have NO affiliations with or involvement in any organization or entity with any financial interest (such as honoraria; educational grants; participation in speakers’ bureaus; membership, employment, consultancies, stock ownership, or other equity interest; and expert testimony or patent‐licensing arrangements), or non‐financial interest (such as personal or professional relationships, affiliations, knowledge, or beliefs) in the subject matter or materials discussed in this manuscript.
